# Air-breathing cathode self-powered supercapacitive microbial fuel cell with human urine as electrolyte

**DOI:** 10.1016/j.electacta.2020.136530

**Published:** 2020-09-01

**Authors:** Carlo Santoro, Xavier Alexis Walter, Francesca Soavi, John Greenman, Ioannis Ieropoulos

**Affiliations:** a Bristol BioEnergy Centre, Bristol Robotics Laboratory, T-Block, UWE, Coldharbour Lane, Bristol BS16 1QY, UK; b Department of Chemistry “Giacomo Ciamician”, Alma Mater Studiorum – Università̀; di Bologna, Via Selmi, 2, 40126, Bologna, Italy; c Biological, Biomedical and Analytical Sciences, UWE, Coldharbour Lane, Bristol, BS16 1QY, UK

**Keywords:** Microbial fuel cell, Supercapacitive mode, Human urine, Galvanostatic discharges, Current/power pulses

## Abstract

In this work, a membraneless microbial fuel cell (MFC) with an empty volume of 1.5 mL, fed continuously with hydrolysed urine, was tested in supercapacitive mode (SC-MFC). In order to enhance the power output, a double strategy was used: i) a double cathode was added leading to a decrease in the equivalent series resistance (ESR); ii) the apparent capacitance was boosted up by adding capacitive features on the anode electrode. Galvanostatic (GLV) discharges were performed at different discharge currents. The results showed that both strategies were successful obtaining a maximum power output of 1.59 ± 0.01 mW (1.06 ± 0.01 mW mL^−1^) at pulse time of 0.01 s and 0.57 ± 0.01 mW (0.38 ± 0.01 mW mL^−1^) at pulse time of 2 s. The highest energy delivered at i_pulse_ equal to 2 mA was 3.3 ± 0.1 mJ. The best performing SC-MFCs were then connected in series and parallel and tested through GLV discharges. As the power output was similar, the connection in parallel allowed to roughly doubling the current produced. Durability tests over ≈5.6 days showed certain stability despite a light overall decrease.

## Highlights

• Air-breathing microbial fuel cell was tested in supercapacitive mode.
• A double cathode addition lead to a decrease in ohmic resistance.
• Apparent capacitance was boosted up by adding capacitive features.
• Maximum power output of 1.59 mW (1.06 mW mL^−1^) was reached at t_pulse_ 0.01s.
• Series and parallel connections improved the galvanostatic discharges.

## Graphical abstract

**Figure:**
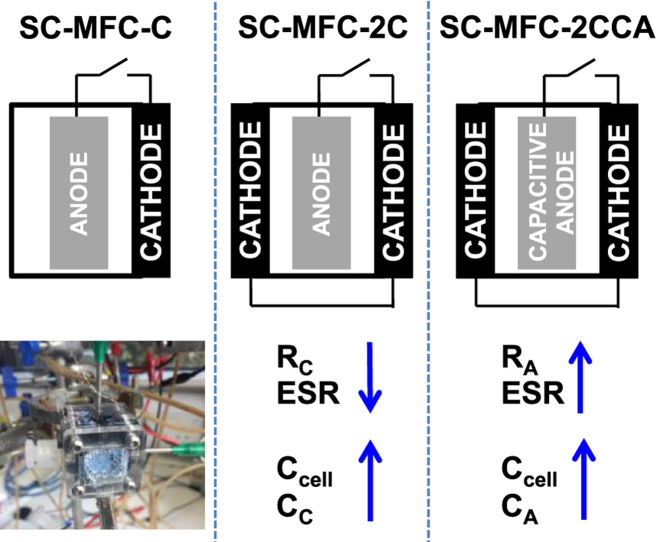

## Abbreviations

AC: activated carbon
BES: Bioelectrochemical system
C_A_: anodic apparent capacitance
C_C_: cathodic apparent capacitance
C_cell_: apparent capacitance
E_pulse_: pulse energy
ESR: equivalent series resistance
GLV: Galvanostatic
i_pulse_: current pulse
MFC: Microbial Fuel Cell
SC-MFC: Supercapacitive Microbial Fuel Cell
OCP: Open Circuit Potential
OCV: Open Circuit Voltage
ORR: oxygen reduction reaction
PGM-free: platinum group metals-free
P_max_: maximum power
P_pulse_: pulse power
PTFE: polytetrafluoroethylene
R_A_: anodic ohmic resistance
R_C_: cathodic ohmic resistance
SC-MFC-C: Supercapacitive Microbial Fuel Cell with single cathode
SC-MFC-2C: Supercapacitive Microbial Fuel Cell with double cathode
SC-MFC-2CCA: Supercapacitive Microbial Fuel Cell with double cathode and capacitive anode
t_pulse_: pulse time
VAP: value-added product
ΔV_capacitive,cell_: voltage variation due to capacitive features
V_max_: maximum voltage after ohmic drop
V_max_: _OC_voltage at open circuit potential
ΔV_ohmic,cell_: vertical voltage drop due to the ohmic losses

## 1 Introduction

Bioelectrochemical systems (BES) are capturing more and more the consideration of the scientific community worldwide for their unique characteristics of transforming the organic compounds into electricity and/or other value-added products (VAPs) [[1], [2], [3], [4], [5]]. Among BESs, microbial fuel cells (MFCs) are of interest because they can remove organics and pollutants from a waste stream and generate simultaneously useful electricity for practical applications [[2], [3], [4], [5]]. Microbial fuel cells have shown the possibilities of degrading the diverse organic compounds varying from single substrate to more complex and variegated wastewaters [6,7]. The fact that MFCs produce rather than consume electricity during their operations makes the technology to be suitable for substituting traditional aerobic wastewater treatment [[2], [3], [4], [5]]. Moreover, since MFCs produce directly electricity through the chemical energy stored in the organic compounds bonds and not indirectly, MFCs can play as competitors to the anaerobic digester technologies in which biogas is created and then transformed to electricity, therefore electricity is produced only indirectly [[2], [3], [4], [5]]. MFCs hold a great potential in the field of wastewater treatment, but are currently limited in terms of industrial scalability, capital expenditure and to a lesser extent, universal applicability (e.g. where there is only solid matter), constraints which have nevertheless been faced by almost all other technologies during their critical development phase.

In order to enter into the market and substitute the existing technologies, MFCs have to: i) reduce the overall cost; ii) increase the cathode reaction rate; iii) improve the power that can be delivered. As MFCs are a low power producing technology, reducing the overall cost is very important. This means that both anode and cathode materials and eventually the membrane/separator have to be durable, reliable and cheap [[8], [9], [10], [11], [12], [13]]. Different anode materials have been investigated with the intention of increasing the biotic/abiotic interphase and therefore enhance the electron transfer from the bacteria to the electrode [14,15]. Among the materials investigated, carbonaceous materials are the most promising [8,9]. Carbon cloth, paper, veil, brush and felt, plain or modified materials have been successfully investigated in MFCs [[16], [17], [18], [19], [20], [21], [22]]. Recently also metallic based materials have been investigated [[23], [24], [25]]. Stainless steel seems to be promising and for large scale applications also cheaper compared to carbonaceous materials [26,27]. Other metals have also been investigated and molybdenum has shown to be a metal increasing the anodic output [28]. Concerning the cathode, substitution of platinum with cheaper and more durable materials is needed [[29], [30], [31]]. The cathode reaction of reducing oxygen is limited by the circum(neutral) electrolyte operating conditions that do not provide high concentration of reactants (e.g. H^+^) [[32], [33], [34]]. Much attention has been devoted to enhance and optimize the oxygen reduction reaction (ORR). As mentioned above, platinum cannot be taken into account due to the high cost and low durability in the presence of anions [35,36] and therefore alternatives have been proposed for accelerating ORR. Carbonaceous materials are suitable to be used as cathode catalysts in bioelectrochemical systems [37,38]. Among them, diverse materials such as carbon black, carbon nanotubes, carbon nanofibers, graphene and activated carbon have been investigated [[39], [40], [41], [42], [43], [44]]. The main characteristics of these catalysts for being successful are: i) high surface area and high content in nitrogen defects on the carbonaceous matrix for enhancing the ORR; ii) high electrical conductivity for enhancing electron transfer; iii) high resistivity to corrosion in polluted and harsh environments. Recently, also platinum group metals-free (PGM-free) catalysts were found to be very active towards ORR in (circum)neutral electrolyte. PGM-free materials are catalysts containing an earth abundant transition metal such as Fe, Co, Mn or Ni [[45], [46], [47]]. High performing PGM-free can be categorized into two main groups; the first one that use catalysts produced through pyrolysis (high temperature treatment in controlled atmosphere) of a nitrogen-rich organic precursor and a metal-containing salt [[48], [49], [50], [51], [52]] and the second one through deposition or impregnation of organic molecules with the metal incorporated into their structure such as porphyrins or phthalocyanines [[53], [54], [55], [56]]. Generally, it was previously found that iron was the most active and efficient [57].

Low power output characterize bioelectrochemical systems that operate in neutral pHs and take advantage of slow biological processes. Therefore, the power management has to be optimised in order to harvest successfully the power/current produced [[58], [59], [60]]. Diverse practical applications have also been implemented starting from robotics with GastroBot [61] and the EcoBot family [[62], [63], [64]]. In order to boost the output of MFCs, the latters are usually coupled with a power management system that contains electronics and supercapacitors or batteries [60]. Lately, the capacitive features of the electrodes have been exploited in supercapacitive bioelectrochemical systems and particularly supercapacitive microbial fuel cell (SC-MFC) [[65], [66], [67], [68], [69], [70], [71]]. Capacitive anodes and cathodes have been extensively investigated in the past 5–10 years [[72], [73], [74], [75], [76], [77], [78], [79]]. Particularly, the electrodes of the BESs were utilized as the electrode of an internal supercapacitor and discharged galvanostatically, like a supercapacitor [80]. High current/power pulses were obtained and the electrodes were self-recharged to the initial voltage value conditions due to the red-ox reactions (oxidation of organics and reduction of oxygen) occurring on the two electrodes [80]. Diverse supercapacitive MFCs were tested in different scale varying from 0.5 mL [81] to 1 L [82,83] in different design configurations. These previous design adopted also diverse fuels operating as the electrolyte of the internal supercapacitor. Human urine as fuel was firstly introduced in 2012 [84] and captured the attention of the scientists worldwide in the past few years due to its unique characteristics and properties that make it suitable to be used widely for BESs [85]. Particularly, human urine is of extreme interests due to the high solution conductivity, which help to decrease the ohmic resistance of the electrolyte [86,87].

In this work, a small-size air-breathing cathode membraneless MFC design is presented and investigated in supercapacitive mode. SC-MFC operated in flow with hydrolysed human urine as fuel and electrolyte. Characteristics parameters such as equivalent series resistance (ESR) and apparent capacitance are measured on the overall SC-MFC and on the single electrode. Once the control SC-MFC was characterized, further improvements have been followed in order to decrease the overall ESR and enhance the apparent capacitance. Moreover, the SC-MFCs were connected in series and parallel and the electrochemical output was investigated. Durability tests of 4000 cycles of discharge and self-recharge were carried out in order to establish the variation in performance, ESR and apparent capacitance over time.

## 2 Materials and method

### 2.1 Microbial fuel cell construction and materials

**Fig. 1:**
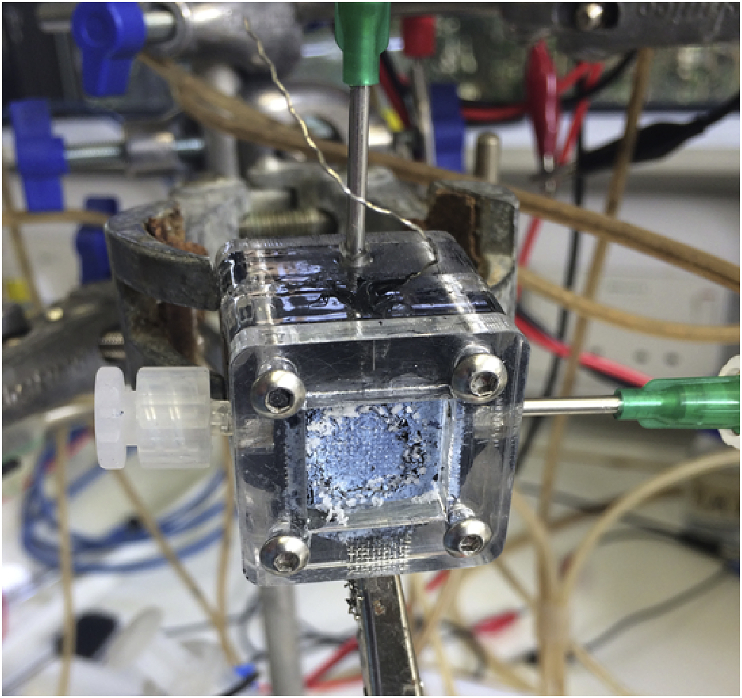
Image of the SC-MFC used in this study.

The SC-MFCs had a cubical shape and was built using Plexiglas pieces that were put together through screws. The cubical had two lateral holes where the cathodes were placed. In the case of the control SC-MFCs, only one cathode was used and the other lateral hole was closed with a laser cut of the shape of the hole. The empty volume was 2 mL but the displacement volume (containing the anode) was 1.5 mL. Two additional holes were built for the inlet and outlet of the electrolyte through the anodic chamber. At last, a hole was made on the top of the SC-MFCs in order to accommodate the reference electrode to be inserted in the chamber. An image of the operating SC-MFC is reported in Fig. 1.

The anode was built using carbon veil. Particularly, a foil of 2 × 5 cm (10 cm^2^) was wrapped into a small cubical shape. The cathode was made with a mixture of activated carbon (AC, 800 m^2^ g^−1^, SK1 P75, CPL Carbon Link, UK) and polytetrafluoroethylene (PTFE) (60% emulsion, Sigma Aldrich) in a ratio in weight of 8 to 2 [88,89]. The mixture was then pressed over a stainless steel mesh using a pin roll. Four layers of PTFE were coated also on the external face (facing atmosphere) of the stainless steel as shown before applying a temperature treatment. The external PTFE diffusion layer was used in order to avoid electrolyte leakage. The SC-MFCs control (in duplicate) named as SC-MFC-C had the anode dimension and composition as described before and one cathode with a geometric surface area of 2.25 cm^2^. The second configuration used was named SC-MFC-2C (in duplicate) and differed from the first configuration by the addition of a second cathode on the opposite side of the MFC. The anode was the same size as the first configuration but the cathode geometric area doubled to 4.5 cm^2^. In the third configuration, a thin layer composed by activated carbon and PTFE was prepared separately and embedded into the anode electrode. The third configuration named as SC-MFC-2CCA (in duplicate) also had two cathodes electrodes with a total geometric area of 4.5 cm^2^. The conditions tested are summarized in Table 1. Experiments were run in duplicates for each condition and the two SC-MFCs were named as A and B. SC-MFCs were firstly individually tested and then the two SC-MFC-2CCAs were studied after being connected in series and in parallel.

**Table 1:** Anode and cathode composition of the three conditions tested.

condition	anode	cathode	acronym
control	carbon veil	1 cathode (2.25 cm^2^)	SC-MFC-C
double cathode	carbon veil	2 cathodes (4.5 cm^2^)	SC-MFC-2C
double cathode & capacitive anode	carbon veil & AC/PTFE	2 cathodes (4.5 cm^2^)	SC-MFC-2CCA

### 2.2 Operating conditions

The MFCs (in duplicate for each studied condition) were inoculated initially with an electrolyte mixture containing 50% in volume of hydrolysed urine and 50% of effluent from well-established and working MFCs fed with hydrolysed urine. The MFCs were connected to an external resistor that was decreased from 5000 Ω to 2500 Ω, 1000 Ω and finally 500 Ω. Once the voltage output was stable, fresh fully hydrolysed urine was used as feedstock. All the SC-MFCs were run in continuous and constant flow of 0.06 mL min^−1^. The urine used had a pH of 9.1 ± 0.1 and a solution conductivity that varied in the range of 26.2 and 30.3 mS cm^−1^. Once the voltage was stable, galvanostatic discharges were done on the MFCs singularly considering anode as negative electrode and cathode as positive electrode of an internal supercapacitor. Once these tests were done, SC-MFC-2CCAs were connected firstly in parallel and then in series and galvanostatic discharges were performed.

### 2.3 Electrochemical operations

Complete galvanostatic (GLV) discharges were done at different current pulses (**i**_**pulse**_). Experiments were done in duplicates. The MFCs were initially left in open circuit conditions for at least 1 h. After 1 h, the voltage of the MFC was stable at a value named as **V**_**max,OC**_. When the GLV started, a vertical drop in the voltage can be noted. The vertical voltage drop corresponded to the ohmic losses (**ΔV**_**ohmic,cell**_) of the system and the MFC reach then a value named **V**_**max**_, which is actually the maximum voltage that the MFC can feature. **V**_**max**_ can be described by eq. (1):

(1) $$V_{max}=\;V_{max,OC}-\;\Delta V_{ohmic,cell}$$

The ohmic losses named also equivalent series resistance (**ESR**) corresponded to the sum of anode electrode ohmic losses, electrolyte ohmic losses and cathode ohmic losses. ESR can be calculated using eq. (2) as the ratio between **ΔV**_**ohmic,cell**_ and **i**_**pulse**_:

(2) $$ESR=\;\frac{\Delta V_{ohmic,cell}}{i_{pulse}}$$

During GLVs, a reference electrode was inserted into the MFC close to the anode electrode. This allowed measuring the anode and cathode potential profile separately. Particularly, also the anode and cathode ohmic resistance named as **R**_**A**_ and **R**_**C**_ respectively, can be calculated. Eq. (3) is used for calculating **R**_**A**_:

(3) $$R_{A}=\;\frac{\Delta V_{ohmic,anode}}{i_{pulse}}$$

Eq. (4) instead is used for calculating **R**_**C**_:

(4) $$R_{C}=\;\frac{\Delta V_{ohmic,cathode}}{i_{pulse}}$$

After reaching V_max_, the voltage continues to decrease and this is due to the electrodes capacitive features. This voltage variation named as capacitive variation (**ΔV**_**capacitive,cell**_) is used for calculating the capacitance of the cell (**C**_**cell**_) following eq. (5):

(5) $$C_{cell}=\;\frac{i_{pulse}}{\frac{dV}{dt}}=\;\frac{i_{pulse}}{s}$$

The slope s is defined as the variation of voltage over time. As done for ESR, also the apparent capacitance can be calculated for anode (C_A_) and cathode (C_C_) electrode respectively. In the case of C_A_ and C_C_, the variation of the potential during the discharge will be considered. C_A_ and C_C_ were calculated according to eq. (6) and eq. (7):

(6) $$C_{A}=\;\frac{i_{pulse}}{\frac{dV_{anode}}{dt}}$$

(7) $$C_{C}=\;\frac{i_{pulse}}{\frac{dV_{cathode}}{dt}}$$

Apparent **C**_**A**_, **C**_**C**_ and **C**_**cell**_ can be correlated as shown in eq. (8):

(8) $$C_{cell}={\left(\frac{1}{C_{A}}+\;\frac{1}{C_{C}}\right)}^{-1}$$

In the calculation of the apparent capacitance, only the initial linear variation of the cell voltage and electrodes potential is considered. For low i_pulse_, the cell voltage and electrodes potential reach a plateau that cannot be accounted for the electrostatic double layer but it can be accounted for by the redox reaction occurring at the interface of the electrodes/electrolyte. Electrostatic and faradaic contributions during galvanostatic discharges were deeply studied in a recent investigation [90].

Power and energy are other parameters that are used for characterizing the system. Particularly, the maximum power (**P**_**max**_) achievable by the system is determined using eq. (9):

(9) $$P_{max}=\;V_{max}\;\times \;i_{pulse}$$

**P**_**max**_ considers the ohmic losses of the system but disregard the capacitive losses. The pulse power (**P**_**pulse**_) delivered in a specific time (**t**_**pulse**_) instead is calculated in eq. (10):

(10) $$P_{pulse}=\;\frac{i_{pulse\;}\int_{0}^{t}Vdt}{t_{pulse}}$$

The energy of the pulse is also calculated considering **P**_**pulse**_ and **t**_**pulse**_ as shown in eq. (11):

(11) $$E_{pulse}=\;P_{pulse\;}\times \;t_{pulse}$$

The power is also expressed in function of the volume (mW mL^−1^) of the MFC that was 1.5 mL.

## 3 Results and discussion

### 3.1 Complete discharges of air-breathing cathode supercapacitive MFC

**Fig. 2:**
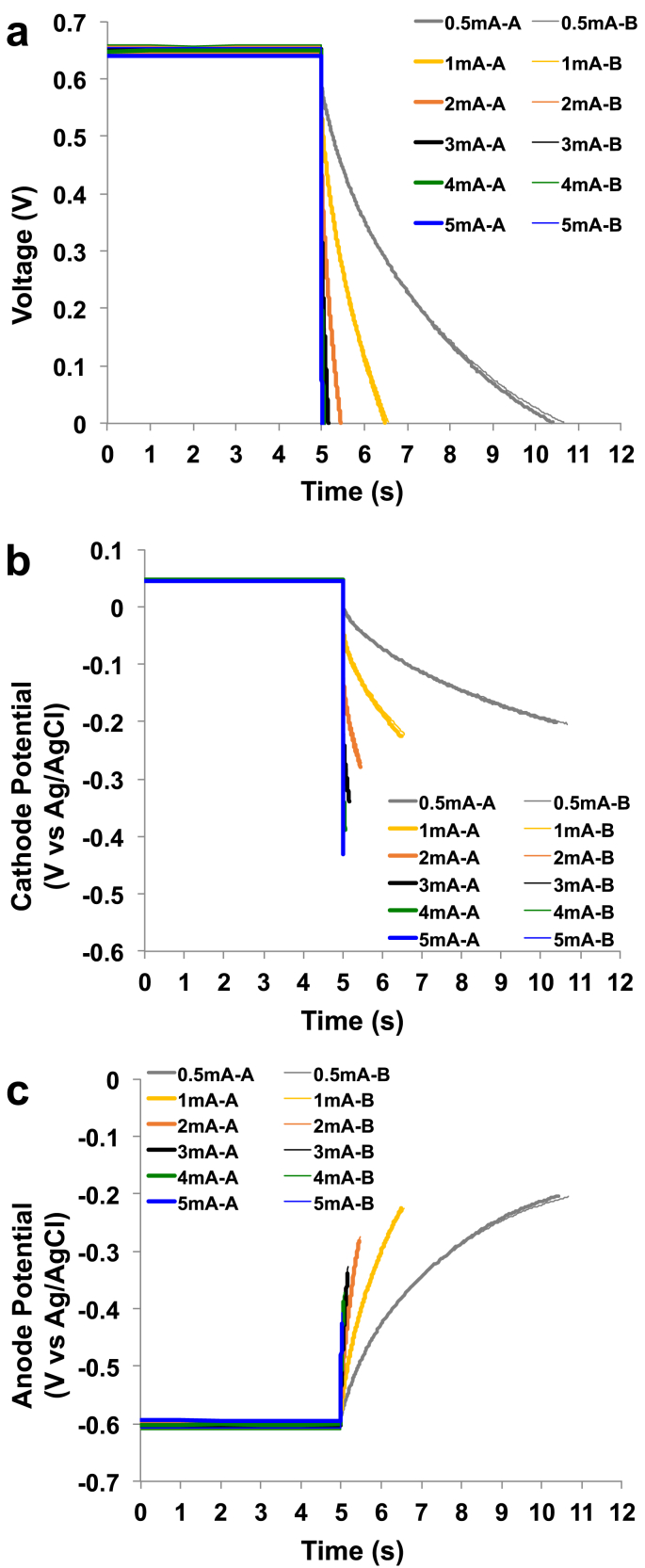
Complete discharges for SC-MFC at different i_pulse_. Overall (a), cathode (b), anode (c) profile during the discharge. A and B are the two separate SC-MFCs.

Complete GLVs were achieved on the SC-MFCs at different i_pulse_ varying from 0.5 mA to 5 mA. SC-MFC voltage trends and cathode and anode potential behaviours were presented in Fig. 2.a, Fig. 2.b and Fig. 2.c respectively. V_max,OC_ was measured to be 650 ± 6 mV and was the difference between the cathode potential contribution (47 ± 2 mV vs Ag/AgCl) and the anode potential contribution (−603 ± 5 mV vs Ag/AgCl). The maximum voltage of an MFC operating with organics and using oxygen as final electron acceptor is ≈ 1100 mV [91], therefore the overpotentials can be quantified in ≈450 mV. At pH 9, organic oxidation involving NADH/NAD^+^ has a potential of ≈ -600 mV vs Ag/AgCl and this value is close to the anodic potential recorded indicating negligible overpotentials. The overall overpotentials can be attributed exclusively to the cathode.

ESR was evaluated to be 87.9 ± 3.2 Ω and the 20% of it was due to the anode (18.1 ± 0.5 Ω) and the remaining 80% due to the cathode (70.1 ± 2.9 Ω). Larger cathode resistance was also measured in previous works underlying the limitation of this particular electrode [92,93]. The time of complete discharges decreased with the increase in i_pulse_. Particularly, t_pulse_ of 5.55 ± 0.13 s, 1.54 ± 0.04 s, 0.47 ± 0.02 s, 0.17 ± 0.02 s, 0.07 ± 0.01 s and 0.03 ± 0.01 s were measured for i_pulse_ of 0.5 mA, 1 mA, 2 mA, 3 mA, 4 mA and 5 mA respectively. The apparent capacitance of the SC-MFC and anode and cathode separately can be found in Table S1. Apparent C_cell_ decreased with the current applied indicating a probable contribution from the red-ox reactions occurring on the electrode and therefore a faradaic component. Except for the highest i_pulse_ tested, the apparent capacitance of the cathode was much higher compared to the one of the anode. This can be expected since carbon veil does not exhibit high surface area and does not possess capacitive features. At the contrary, the cathode is composed by AC, which has high surface area and good capacitive features.

### 3.2 Effect of cathode doubling on the galvanostatic discharges

**Fig. 3:**
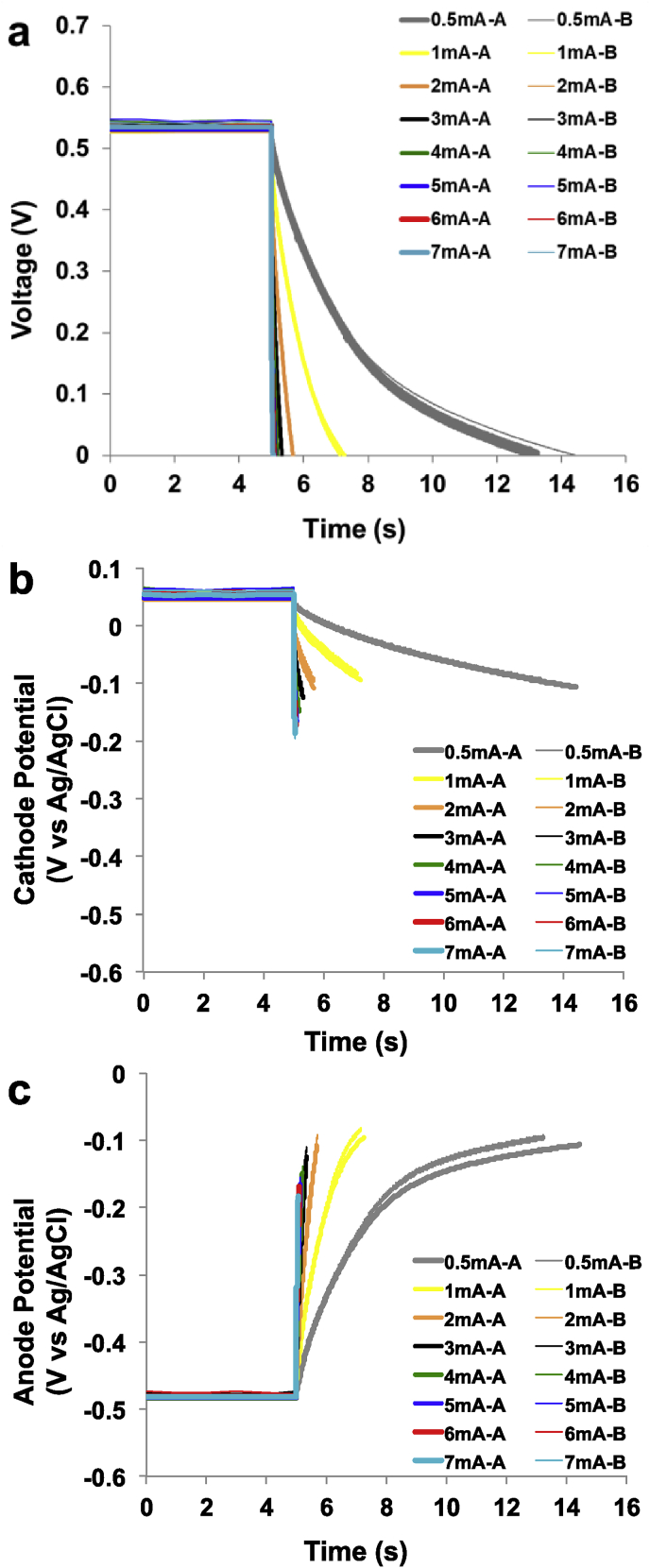
Complete discharges for SC-MFC-2C at different i_pulse_. Overall (a), cathode (b), anode (c) profile during the discharge. A and B are the two separate SC-MFCs.

As the cathode of SC-MFC-Cs showed high R_C_ contributing to over 80% of the overall ESR, the first action done for improving the performance was to reduce R_C_. In order to achieve that, the cathode area was doubled and particularly at both side of the structure, a cathode was inserted. Overall voltage trends and cathode and anode potential behaviors of SC-MFC-2C during GLV discharges were presented in Fig. 3.a, Fig. 3.b and Fig. 3.c, respectively.

This configuration lead to a decrease in cell OCV to 536 ± 6 mV that was ≈114 mV lower compared to the one recorded for SC-MFC-C that was 650 ± 6 mV. In order to understand the cause of this decrease, also the anode and cathode potential were examined. The cathode potential measured similarly as SC-MFC-C (+55 ± 7 mV vs Ag/AgCl), instead the anode had much higher potential. In fact, the anode potential measured −480 ± 3 mV vs Ag/AgCl with a decrease of roughly 120 mV compared to the control. This increase in the anode potential might be due to the addition of the second cathode and therefore the anode resulted to be more exposed to oxygen penetrating through the cathode and negatively affecting the anaerobic environment. Despite this negative aspect, during GLV discharges, showed a significant decrease in R_C_ and consequently ESR. ESR was measured as 40.0 ± 2.0 Ω with R_A_ being 16.7 ± 0.5 Ω and RC being 22.2 ± 1.7 Ω. R_A_ was similar as before while R_C_ was a third than SC-MFC-C thus indicating that this strategy was advantageous. Despite lower OCV, the SC-MFC-2Cs could be completely discharged at higher i_pulse_ (up to 7 mA). At i_pulse_ of 0.5 mA, t_pulse_ was 8.84 ± 0.59 s, which was almost double compared to SC-MFC-C. At i_pulse_ of 1 mA, t_pulse_ was 2.20 ± 0.05 s. In this case, the t_pulse_ was lower compared to SC-MFC-C considering the same i_pulse_. Therefore the advantage of lower ESR was only notable at lower i_pulse_, at higher i_pulse_ instead this advancement disappeared. The overall apparent capacitance of the SC-MFC-2C as well as the anode and the cathode separately are presented in Table S2. Despite lower t_pulse_, the lower OCV and ESR allowed for a higher apparent capacitance. Apparent cathode capacitance was higher compared to anode capacitance. As the cathode doubled in size, the apparent capacitance of the cathode was always higher at every i_pulse_ investigated.

### 3.3 Effect of addition of supercapacitive features into the anode

**Fig. 4:**
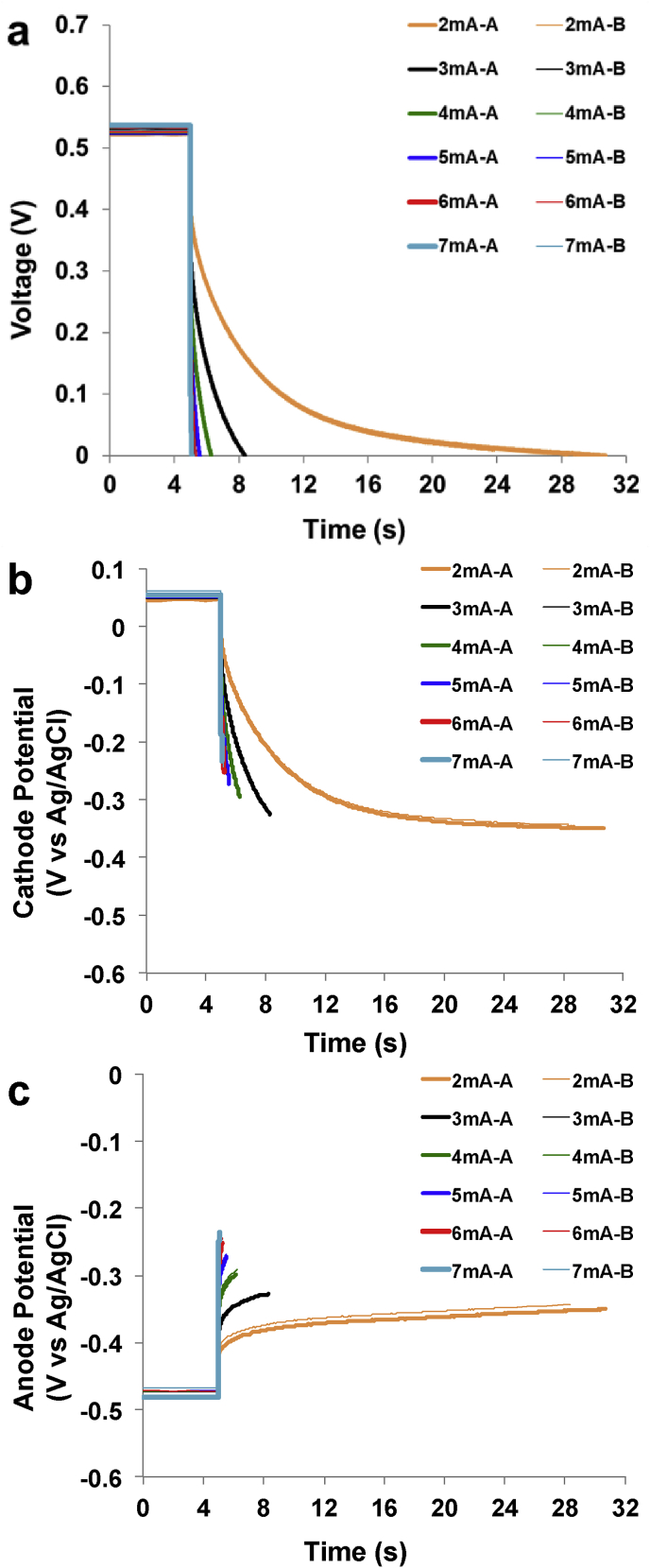
Complete discharges for SC-MFC-2CCA at different i_pulse_. Overall (a), cathode (b), anode (c) profile during the discharge. A and B are the two separate SC-MFCs.

The cathode ohmic resistance (R_C_) was diminished by adding the second cathode as discussed in the previous section. The next strategy adopted to enhance the performance was to add a supercapacitive layer made by a mixture of AC and PTFE and embedded within the carbon veil anode. Complete GLV discharges for SC-MFC-2CCA were presented in Fig. 4.

The OCV remained comparable compared to SC-MFC-2C measuring 527 ± 7 mV. In this case, the overall ESR increased measuring 49.03 ± 1.08 Ω with R_A_ being 26.33 ± 2.20 Ω and R_C_ being 22.71 ± 2.39 Ω. R_C_ remained almost stable compared to the previous set of SC-MFCs while R_A_ increased by roughly 10 Ω. This might be due to a worse connection or to the presence of PTFE that decreased the electrode conductivity. Despite higher ESR, t_pulse_ for complete discharge increased for any i_pulse_ investigated (Table S3). Particularly, at i_pulse_ of 2 mA, t_pulse_ was 24.54 ± 1.63 s, which was much higher compared to SC-MFC-C and SC-MFC-2C. At the higher i_pulse_ investigated (7 mA), t_pulse_ was 0.12 ± 0.03 s (Table S3). Overall apparent capacitance values and the one of each single electrode are reported in Table S3. For low i_pulse_ (2 mA), only the initial linear part was considered in the apparent capacitance calculation. Also in this case, the apparent capacitance increased with the decreasing i_pulse_ and the values recorded varied between 36.37 ± 0.31 mF (i_pulse_ 2 mA) and 4.86 ± 0.82 mF (i_pulse_ 7 mA). With the addition of capacitive features on the anode, its apparent capacitance improved significantly. In this case, the apparent capacitance was higher compared to the one of the cathode (Table S3) being beneficial for the overall capacitance.

### 3.4 Effect of series and parallel connection during the galvanostatic discharges

**Fig. 5:**
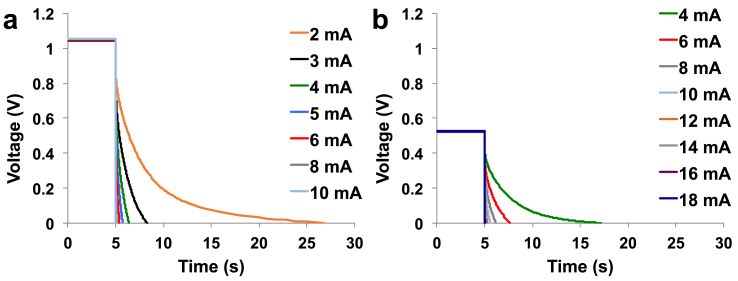
Complete discharges at different i_pulse_ for SC-MFC-2CCA connected in series (a) and in parallel (b).

In the previous paragraph, the overall electrochemical performance during galvanostatic discharges were improved by firstly doubling the cathode area and then by adding capacitive features within the anode electrode. The SC-MFC-2CCAs (double cathode and capacitive anodes) were then connected firstly in parallel and then in series and complete GLV discharges were conducted. Unfortunately, the trend of each single electrode using a reference electrode could not be monitored due to the fact that the two SC-MFCs were hydraulically disconnected. Complete discharges with SC-MFC-2CCAs connected in series are shown in Fig. 5.a. Instead, complete discharges with SC-MFC-2CCAs connected in parallel are shown in Fig. 5.b.

Parameters of interests such as i_pulse_, ESR, discharge time and apparent capacitance are summarized in Table 2. Concerning the series connection, OCV almost doubled measuring 1050 ± 9 mV and ESR was 75.0 ± 0.9 Ω. Discharge time varied between 0.03 s (i_pulse_ 10 mA) and 21.77 s (i_pulse_ 2 mA). Apparent capacitance also varied between 0.72 mF (i_pulse_ 10 mA) and 16.15 mF (i_pulse_ 2 mA). The connection series enhanced the operating voltage but did not contribute on increasing the time of discharge. Parallel connection led to an OCV of 525 ± 5 mV and an ESR of 22.7 ± 0.4 Ω. Discharge time increased substantially at lower current pulse. The apparent capacitance improved importantly achieving the higher value of 62.99 mF (i_pulse_ 4 mA).

**Table 2:** ESR, discharge time and apparent capacitance for SC-MFC-2CCAs connected in series or parallel.

Series		discharge	apparent	Parallel		discharge	apparent
i_pulse_	ESR	time	C_tot_	i_pulse_	ESR	time	C_tot_
mA	Ω	s	mF	mA	Ω	s	mF
2	73.3	21.77	16.15	4	23	12.20	62.99
3	75.6	3.31	12.17	6	23	2.71	42.60
4	75.9	1.39	7.48	8	22.8	1.17	27.51
5	74.9	0.75	5.53	10	22.7	0.58	19.46
6	75.1	0.40	3.99	12	22.9	0.27	13.02
7	74.3	0.23	3.07	14	22.8	0.13	9.15
8	74.7	0.12	2.09	16	22.7	0.06	5.84
10	74.4	0.03	0.72	18	22.6	0.02	3.57
	75.0 ± 0.9				22.7 ± 0.4		

The Ragone plots related to SC-MFC-Cs, SC-MFC-2Cs and SC-MFC-2CCAs are reported in Figure S1.a. The Ragone plots related to SC-MFC-2CCAs connected in series and parallel are reported in Figure S1.b. The Ragone plots are used to compare the energy and power output for each type of SC-MFC. For each system, the highest energy was delivered at the lowest current. The energy delivered at i_pulse_ equal to 2 mA by SC-MFC-2CCAs was 3.3 ± 0.1 mJ.

It can be seen that SC-MFC-2C is performing better than SC-MFC-C for t_pulse_ lower than 1 s but for higher time they performed similarly. This might be due to the fact that SC-MFC-2C had lower ESR but also lower OCV. Interestingly, the best performance were achieved by SC-MFC-2CCA for t_pulses_ higher than 0.5 s. At low t_pulse_, the low OCV and its ESR affected negatively the performance of SC-MFC-2CCA. At higher t_pulse_, the faradaic contribution allowed it to achieve superior performance. The connection in series and in parallel brought to similar curves on the Ragone plots (Figure S1.b) with much superior performance compared to the single SC-MFC-2CCA indicating that the stacking in series or in parallel is a beneficial way for improving the output.

### 3.5 Power curves

**Fig. 6:**
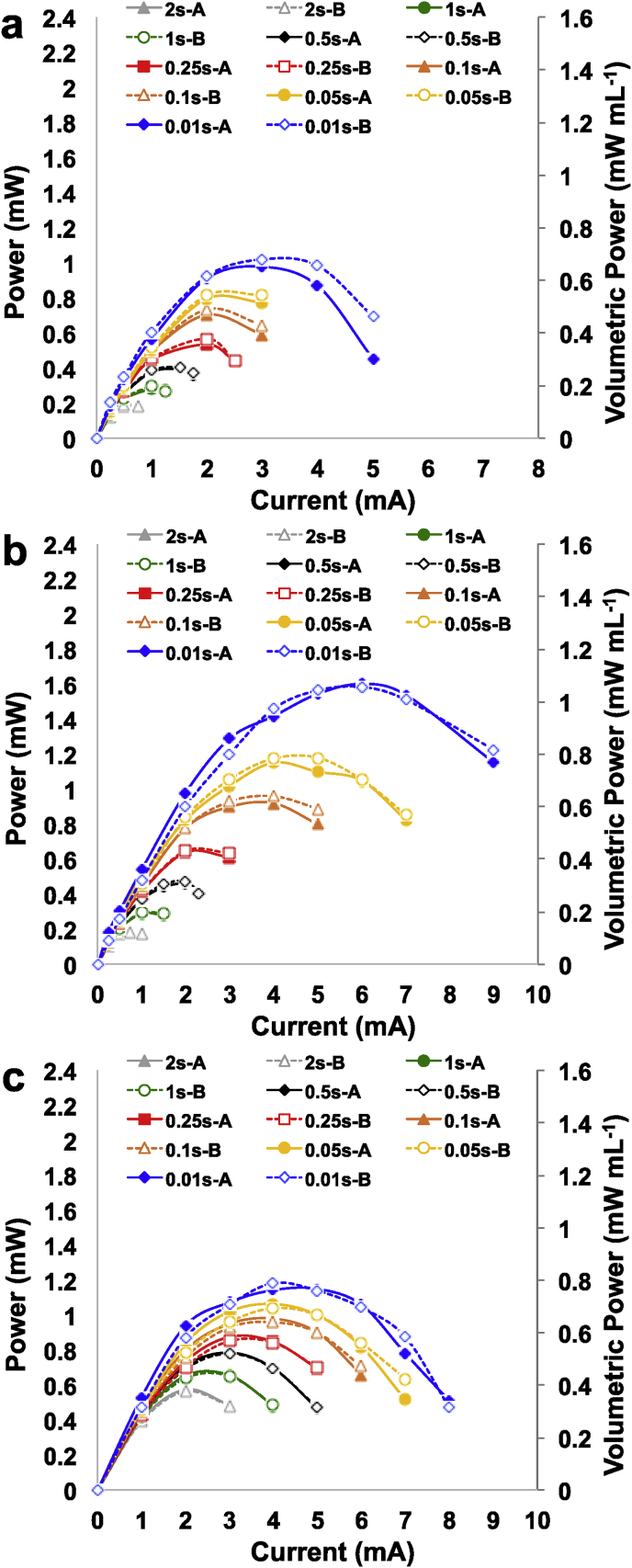
Power curves for SC-MFC-C (a), SC-MFC-2C (b) and SC-MFC-2C-CA (c) considering t_pulse_ of 2 s, 1 s, 0.5 s, 0.25 s, 0.1 s, 0.05 s and 0.01 s. A and B are the two separate SC-MFCs.

Power curves for SC-MFC-C (Fig. 6a), SC-MFC-2C (Fig. 6b) and SC-MFC-2CCA (Fig. 6c) at different t_pulses_ were presented. Higher power was obtained by SC-MFC-2C at shorter t_pulse_ with values recorded of 1.59 ± 0.01 mW (1.06 ± 0.01 mW mL^−1^) and 1.17 ± 0.02 mW (0.78 ± 0.01 mW mL^−1^) at discharge time of 0.01 s and 0.05 s respectively. At t_pulse_ higher than 0.25 s, despite higher ESR and similar OCV, the higher power was achieved by SC-MFC-2CCA because of its higher apparent capacitance. Particularly, the peak of power recorded for SC-MFC-2CCA at t_pulse_ of 0.25 s, 0.5 s, 1 s and 2 s was 0.86 ± 0.02 mW (0.58 ± 0.01 mW mL^−1^), 0.78 ± 0.01 mW (0.52 ± 0.01 mW mL^−1^), 0.65 ± 0.00 mW (0.44 ± 0.00 mW mL^−1^) and 0.57 ± 0.01 mW (0.38 ± 0.01 mW mL^−1^), respectively.

**Fig. 7:**
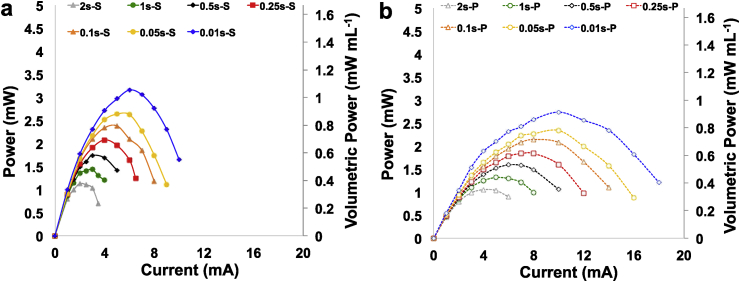
Power curves for two SC-MFC-2C–CAs connected in series (a) and in parallel (b) considering t_pulse_ of 2 s, 1 s, 0.5 s, 0.25 s, 0.1 s, 0.05 s and 0.01 s.

The power curves obtained at different t_pulse_ for SC-MFC-2CCA connected in series and in parallel are instead shown in Fig. 7a and 7.b respectively. The peaks of power curves are reported in Table S4. Generally, the peak of power is greater for the series connection despite it occurring at lower current. As expected, the current delivered by the parallel connection of the two SC-MFC-2CCAs is greater compared to the series connection of the latters.

### 3.6 Durability tests

**Fig. 8:**
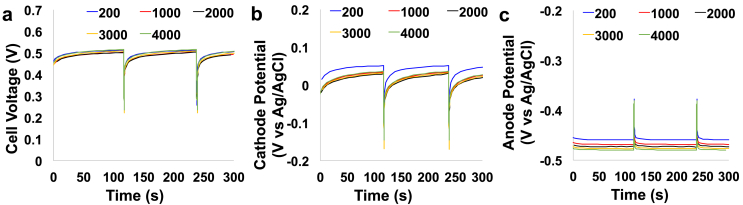
Durability test of a single SC-MFC-2CCA for 4000 cycles of discharge at i_pulse_ of 2 mA and self-recharge. Overall voltage (a), cathode potential (b) and anode potential (c) recorded for cycle 200, 1000, 2000, 3000 and 4000.

Longevity tests of discharge self-recharge were conducted in order to study the durability of the system. Discharges were done delivering an i_pulse_ of 2 mA for a t_pulse_ of 1 s. Discharges were followed by a period of rest of 120 s in which the cell was disconnected and no current was flowing within the circuit. In this case, the electrodes were self-recharged due to the anaerobic/aerobic environments occurring in the proximity of the two electrodes, anode and cathode respectively. 4000 cycles of discharges/self-recharges were conducted for a lapse of time equivalent to ≈135 h (≈5.6 days). The overall voltage trend during the durability test is reported in Figure S2.a while the separate anode and cathode potential trends are reported in Figure S2.b and S2.c, respectively. In order to study the durability of the system, cycle number 200, 1000, 2000, 3000 and 4000 were separate from the overall test and reported in Fig. 8. Particularly, cell voltage, cathode potential and anode potential at these specific cycles were reported in Fig. 8.a, Fig. 8.b and Fig. 8.c respectively. Data measured for OCV, OCP_a_, OCP_c_, ESR, R_A_, R_C_, C_cell_, C_A_ and C_C_ recorded for cycle 200, 1000, 2000, 3000 and 4000 were also reported in Table S5. OCV varied slightly between 501 mV (cycle 2000) and 516 mV (cycle 4000) (Fig. 8a). The cathode OCP tended to decrease as well except for the last cycle where it was slightly higher than the value recorded after 3000 cycles (Fig. 8b). The anodic OCP tended to decrease over the 4000 cycles varying from an initial value of −459 mV (vs Ag/AgCl) to a value of −480 mV (vs Ag/AgCl) (Fig. 8c). From these measurements, it seems that both the anodic and cathodic potentials were decreasing over time. This can be explained by the biofilm formation and biofouling occurring on the cathode electrodes in a short amount of time as previously reported [[94], [95], [96]]. This biofilm/biofouling layer might have lowered the oxygen penetration within the anodic chamber reducing the negative effect on the anode, which became more negative over time due to more established anaerobic environments. Better biofilm adaptation and better anode colonisation might also be important points to consider.

ESR values increased from 51.4 Ω (cycle 200) to 55.7 Ω (cycle 3000) and the slightly decrease in the last cycle (52.9 Ω) (Table S5). The anodic resistance increased over the cycles as well as the cathodic one except the last cycle in which a decrease of 3.4 Ω was measured (Table S5). In parallel, also the apparent capacitance measured for the entire cell decreased over the first 3000 cycles and then slightly increased at cycle 4000 (Table S5). The anodic apparent capacitance decreased over the cycles while the cathodic apparent capacitance followed the trend of the overall apparent capacitance. Generally, it can be notice a slight decrease in ESR and apparent capacitance underlining a slow deterioration of the operating conditions. Interestingly, it can be noticed a decrease in performance around 3500 cycles (Figure S2.a) and this was probably due to the formation/creation of an air-bubble inside the anodic chamber and this might have decreased the anode area exposed to the electrolyte or temporarily negatively affect the anaerobiosis condition within the anode electrode. An increase in the anode potential can be in fact noticed around cycle 3500. Once the problem was identified, the operator removed the bubble and the condition re-established similar to previous conditions. Actually, the removal of the air-bubble enhanced the cathode performance which ESR and apparent capacitance increased during cycle 4000. This was beneficial also to the overall SC-MFC, which recovered part of its losses.

### 3.7 Overview of the current study

In this study, a small air-breathing cathode membraneless MFC with a displacement volume of 1.5 mL was operated in supercapacitive mode. Initially, it was tested using the same materials usually adopted for MFCs operating in standard mode under a constant load. In fact, carbon veil anode and activated carbon-based cathode were adopted. ESR was relatively high mainly due to the cathode electrode and the capacitance of the SC-MFC was very low especially due to the poor capacitance of the carbon veil. In order to boost the performance up, a second cathode was accommodated and a capacitive layer was added to the anode electrode. This double variation led to a decrease in the cathode resistance and therefore overall ESR and to an enhancement of the anode apparent capacitance and therefore the overall SC-MFC apparent capacitance. Unfortunately, both variations had their own negative effect. The addition of the second cathode decreased the SC-MFC OCV probably influencing negatively the anaerobic environment of the anode. The addition of the capacitive features on the anode increased the anodic and overall resistance. It was shown that at shorter discharge time, SC-MFC with double cathode was the best performing with the highest peak of power recorded in this study of 1.59 ± 0.01 mW (1.06 ± 0.01 mW mL^−1^). At longer discharge time, SC-MFC with double cathode and modified anode had the higher power produced (0.57 ± 0.01 mW corresponding to 0.38 ± 0.01 mW mL^−1^) and higher apparent capacitance. Despite being pulsed power and not continuous, the output is more than one order of magnitude higher compared to similar MFCs operating in continuous mode. The decrease in the reactor size compared to previously presented literature showed an increase in power produced [97,98]. As the reactor become smaller, electrode area to volume ratio is optimised, less planktonic and competitive bacteria are present into the systems and distances between electrodes decreases affecting positively the ohmic resistances that are reduced.

At lower discharge current, the faradaic contribution became predominant in agreement with previously presented results [80,90]. Another strategy for enhancing the electrochemical performance is to connect different SC-MFC in series or in parallel. Series connection allows boosting the operating voltage. Parallel connection instead is necessary to improve current generated. The connection in Series or Parallel of two SC-MFCs led to similar power output despite the series connection being slightly more performing. Similar powers generated were obtained even if the connection in parallel allowed reaching almost double current compared to the series connection. Durability tests showed that the system was robust over 4000 cycles despite a light decrease in the parameters of interest.

## 4 Conclusions

Membraneless SC-MFC with air-breathing cathode was tested in supercapacitive mode. Hydrolysed human urine was used as feeding as well as electrolyte. Control SC-MFC having carbon veil anode and AC/PTFE cathode was subject to galvanostatic discharges. The performance was improved by doubling the cathode leading to a diminishment of the ESR and improvement in the apparent capacitance. The performance was improved even more by embedding AC-based capacitive features in the anode electrode. Maximum power output obtained was 1.59 ± 0.01 mW (1.06 ± 0.01 mW mL^−1^) at t_pulse_ of 0.01 s and 0.57 ± 0.01 mW (0.38 ± 0.01 mW mL^−1^) at t_pulse_ of 2 s. The apparent capacitance increased significantly with the decrease of discharge current applied indicating the presence of the faradaic component during the discharge. The higher apparent capacitance recorded in this work considering the initial linear voltage decrease was 36.37 ± 0.31 mF. Connection in series and parallel between different SC-MFC help improve the performance with the series connection enhancing the overall voltage and the parallel connection instead increasing the current delivered. SC-MFC was also discharged and self-recharged for 4000 cycles showing robustness. Compared with previous work, reducing the size of the reactor clearly boosts the volumetric power output.

## Credit author statement

Carlo Santoro: Conceptualization, Methodology, Data curation, Analysis and Interpretation, Investigation, Writing-Original draft preparation, Xavier Alexis Walter: Methodology, Investigation, Data curation, Analysis and Interpretation, Writing- Reviewing. Francesca Soavi: Data Analysis and Interpretation, Writing-Reviewing and Editing, John Greenman: Supervision, Writing-Reviewing and Editing, Ioannis Ieropoulos: Supervision, Funding acquisition, Writing-Reviewing and Editing.

## Declaration of competing interest

The authors declare that they have no known competing financial interests that could have appeared to influence the work reported in this paper.
