# Supplementary material for: Air-breathing cathode self-powered supercapacitive microbial fuel cell with human urine as electrolyte
Source: Electrochim Acta. 2020 Sep 1;353:136530. doi: 10.1016/j.electacta.2020.136530 (PMC7430050; doi:10.1016/j.electacta.2020.136530)
Supplement: Multimedia component 1 [file mmc1.docx]

**Air-breathing cathode self-powered supercapacitive microbial fuel cell with human urine as electrolyte**

*Carlo Santoro^1,+^, Xavier Alexis Walter^1^, Francesca Soavi^2^, John Greenman^1,3^, **Ioannis Ieropoulos^1^

^1^ Bristol BioEnergy Centre, Bristol Robotics Laboratory, T-Block, UWE, Coldharbour Lane, Bristol BS16 1QY, UK

^2^ Department of Chemistry “Giacomo Ciamician”, Alma Mater Studiorum – Università di Bologna, Via Selmi, 2, 40126 Bologna, Italy

^3^ Biological, Biomedical and Analytical Sciences, UWE, Coldharbour Lane, Bristol, BS16 1QY, UK

^+^ Currently: Department of Chemical Engineering and Analytical Science, The University of Manchester, The Mill, Sackville Street, M13AL Manchester, UK

*Corresponding authors:

* Carlo Santoro. [carlo.santoro830@gmail.com](mailto:carlo.santoro830@gmail.com)

** Ioannis Ieropoulos. ioannis.ieropoulos@brl.ac.uk

**Table S1.** Apparent overall capacitance and anode and cathode capacitance for SC-MFC-C for i_pulse_ varying between 0.5 mA and 5 mA

|  |  | **Apparent Capacitance** | | |
| --- | --- | --- | --- | --- |
| **i_pulse_** | **t_pulse_** | **C_cell_** | **C_A_** | **C_C_** |
| **mA** | **s** | **mF** | **mF** | **mF** |
| **0.5** | 5.55±0.13 | 4.61±0.07 | 7.14±0.12 | 13.06±0.15 |
| **1** | 1.54±0.04 | 2.74±0.03 | 4.27±0.04 | 7.64±0.10 |
| **2** | 0.47±0.01 | 1.94±0.01 | 3.19±0.01 | 4.92±0.07 |
| **3** | 0.17±0.01 | 1.32±0.01 | 2.35±0.01 | 2.98±0.09 |
| **4** | 0.07±0.01 | 0.94±0.04 | 1.83±0.01 | 1.92±0.15 |
| **5** | 0.03±0.01 | 0.61±0.09 | 1.33±0.09 | 1.07±0.23 |

**Table S2.** Apparent overall capacitance and anode and cathode apparent capacitance for SC-MFC-2C for i_pulse_ varying between 0.5 mA and 7 mA.

|  |  | **Apparent Capacitance** | | |
| --- | --- | --- | --- | --- |
| **i_pulse_** | **t_pulse_** | **C_cell_** | **C_A_** | **C_C_** |
| **mA** | **s** | **mF** | **mF** | **mF** |
| **0.5** | 8.84±0.57 | 5.62±0.12 | 7.48±0.23 | 24.72±0.51 |
| **1** | 2.20±0.05 | 4.48±0.15 | 5.95±0.22 | 18.37±0.01 |
| **2** | 0.69±0.01 | 2.99±0.01 | 3.98±0.01 | 12.00±0.14 |
| **3** | 0.33±0.00 | 2.38±0.03 | 3.24±0.05 | 8.91±0.01 |
| **4** | 0.20±0.00 | 2.05±0.01 | 2.88±0.02 | 6.98±0.03 |
| **5** | 0.13±0.01 | 1.89±0.04 | 2.72±0.07 | 6.07±0.11 |
| **6** | 0.09±0.00 | 1.68±0.01 | 2.48±0.00 | 5.08±0.08 |
| **7** | 0.06±0.00 | 1.51±0.01 | 2.29±0.02 | 4.28±0.11 |

**Table S3.** Apparent overall capacitance and anode and cathode capacitance for SC-MFC-2CCA for i_pulse_ varying between 2 mA and 7 mA.

|  |  | **Apparent Capacitance** | | |
| --- | --- | --- | --- | --- |
| **i_pulse_** | **t_pulse_** | **C_cell_** | **C_A_** | **C_C_** |
| **mA** | **s** | **mF** | **mF** | **mF** |
| **2** | 24.54±1.63 | 36.37±0.31 | 208.71±2.57 | 43.80±0.23 |
| **3** | 3.34±0.02 | 27.59±0.03 | 142.70±3.39 | 34.21±0.04 |
| **4** | 1.29±0.01 | 16.43±0.18 | 70.96±2.29 | 21.30±0.10 |
| **5** | 0.58±0.01 | 10.96±0.22 | 41.1±1.66 | 14.94±0.18 |
| **6** | 0.24±0.02 | 6.94±0.32 | 22.71±1.67 | 9.92±0.33 |
| **7** | 0.12±0.03 | 4.86±0.82 | 14.70±3.08 | 7.27±1.02 |

**Table S4.** Peak of power curves for SC-MFC-2CCA connected in series and parallel for different t_pulse_

|  | **Series** | | **Parallel** | |
| --- | --- | --- | --- | --- |
| **s** | **mW** | **mW mL^-1^** | **mW** | **mW mL^-1^** |
| **0.01** | 3.16 | 1.05 | 2.74 | 0.91 |
| **0.05** | 2.64 | 0.88 | 2.34 | 0.78 |
| **0.1** | 2.38 | 0.79 | 2.09 | 0.70 |
| **0.25** | 2.08 | 0.69 | 1.84 | 0.61 |
| **0.5** | 1.75 | 0.58 | 1.59 | 0.53 |
| **1** | 1.44 | 0.48 | 1.32 | 0.44 |
| **2** | 1.13 | 0.38 | 1.06 | 0.35 |

**Figure S1.** Ragone plot for SC-MFC-C, SC-MFC-2C and SC-MFC-2CCA (a). Ragone plot comparing SC-MFC-2CCA and the duplicate connected in series and parallel (b).


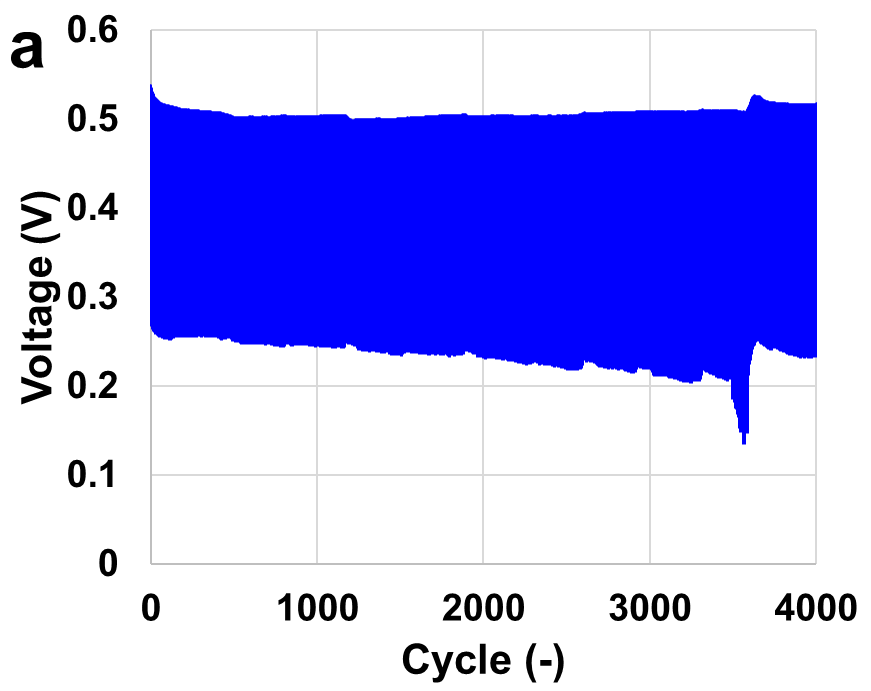

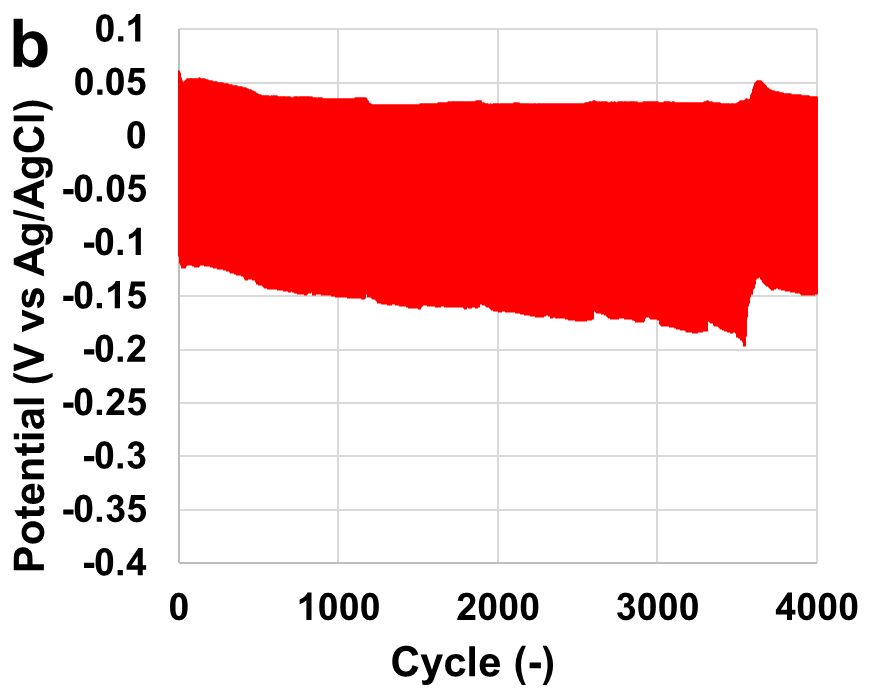

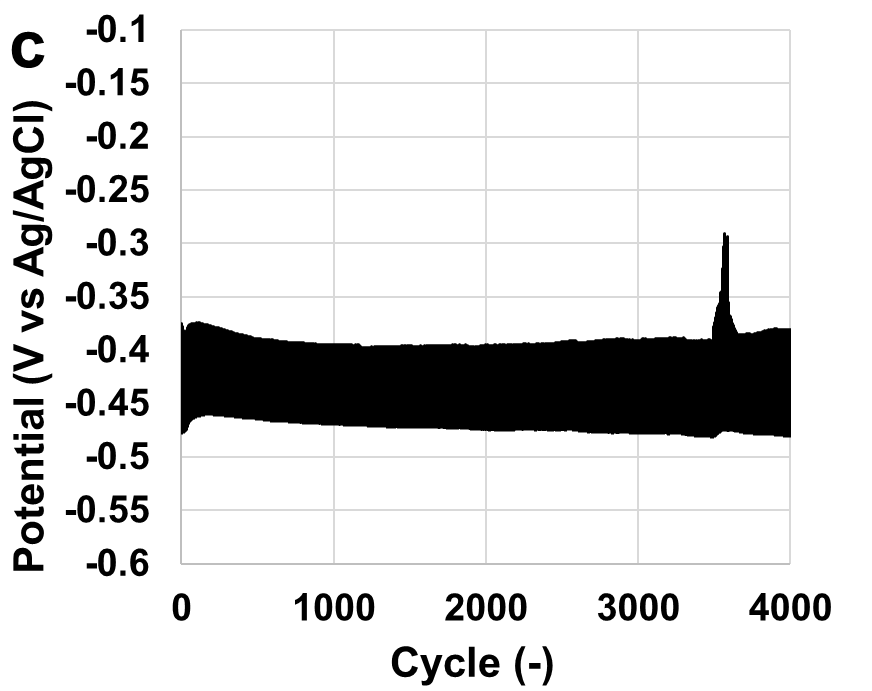


**Figure S2.** Durability test of a single SC-MFC-2CCA for 4000 cycles of discharge (1 s at 2 mA) followed by 120 s of self-recharge

**Table S5.** Data measured for OCV, OCP_a_, OCP_c_, ESR, R_A_, R_C_, C_cell_, C_A_ and C_C_ recorded for cycle 200, 1000, 2000, 3000 and 4000.

|  | **OCV** | **OCP_a_** | **OCP_c_** |
| --- | --- | --- | --- |
| **cycle** | **mV** | **mV vs Ag/AgCl** | |
| **200** | 510 | -459 | 51 |
| **1000** | 503 | -469 | 34 |
| **2000** | 501 | -472 | 29 |
| **3000** | 508 | -477 | 32 |
| **4000** | 516 | -480 | 36 |
|  | **ESR** | **R_A_** | **R_C_** |
| **cycle** | **Ω** | **Ω** | **Ω** |
| **200** | 51.4 | 25.8 | 26 |
| **1000** | 51.5 | 26.1 | 26.1 |
| **2000** | 52.7 | 26.4 | 27.2 |
| **3000** | 55.7 | 28.1 | 27.6 |
| **4000** | 52.9 | 28.7 | 24.2 |
|  | **C_cell_** | **C_A_** | **C_C_** |
| **cycle** | **mF** | **mF** | **mF** |
| **200** | 7.91 | 24.44 | 11.67 |
| **1000** | 7.8 | 27.13 | 10.94 |
| **2000** | 7.48 | 26.4 | 10.44 |
| **3000** | 7 | 23.4 | 9.94 |
| **4000** | 7.13 | 20.19 | 10.93 |
